# Pan-genomics of polyploid crops: from complexity to breeding

**DOI:** 10.3389/fpls.2026.1800432

**Published:** 2026-03-31

**Authors:** Yogesh Dashrath Naik, Mahendar Thudi, Nadia Kamal, Ashley N. Egan, Madhusudhana R. Janga, Naveen Puppala

**Affiliations:** 1 Agricultural Science Center, New Mexico State University, Clovis, NM, United States; 2 Centre for Crop Health and School of Agriculture and Environmental Science, University of Southern Queensland, Toowoomba, QLD, Australia; 3 School of Life Sciences, Technical University of Munich, Freising, Germany; 4 Department of Biology, Utah Valley University, Orem, UT, United States; 5 Institute of Genomics for Crop Abiotic Stress Tolerance, Department of Plant and Soil Science, Texas Tech University, Lubbock, TX, United States

**Keywords:** genome complexity, pan-genome, polyploidy, sequencing, structural variations

## Abstract

Improving crop productivity is essential to ensure global food security in the context of climate change and an increasing global population. Over the past few decades, sequencing has significantly expanded our ability to explore complex genomes. However, the inherent genomic complexity of many plant species, characterized by large genome sizes, high repetitiveness and polyploidy, continues to pose significant challenges for genome assembly and the accurate detection of genetic variation. In particular, structural variations, which are key drivers of trait diversity and genome evolution, are often underrepresented in analyses based on a single linear reference genome due to reference bias. To overcome these constraints, the concept of the pan-genome has emerged. By capturing both core and variable sequences/genes across individuals of a species or genus, pan-genomes offer a more comprehensive representation of genomic diversity. This approach has been successfully implemented in many major crops, including complex polyploids like wheat, peanut, cotton, oat and mustard, and is increasingly contributing to ecological and evolutionary research. This review provides an overview of the development of pan-genome approaches and their application in understanding plant genome complexity, with a focus on trait discovery and modern breeding strategies. It also addresses current challenges and outlines future directions for leveraging pan-genomic resources in crop improvement and biodiversity conservation. In addition, the emerging need for polyploid-aware pan-genome frameworks that explicitly resolve subgenomes, homoeolog dosage, and homoeologous exchange is emphasized to enable breeder-ready applications.

## 1 Introduction

Polyploid crops such as bread wheat (*Triticum aestivum*), potato (*Solanum tuberosum*), cotton (*Gossypium hirsutum*), peanut (*Arachis hypogaea*), canola (*Brassica napus*), oat (*Avena sativa*), and *Brassica* vegetables underpin global food security. These crops are broadly classified as allopolyploids, which possess chromosome sets derived from different species, and autopolyploids, which contain multiple chromosome sets from the same species. In allopolyploids, the distinct parental genomes (subgenomes) may undergo homoeologous exchange (HE), leading to genomic rearrangements and novel allelic combinations that influence trait variation and adaptation. Polyploid genomes are inherently complex, often characterized by large genome sizes and high repeat content, which together complicate genome assembly and downstream analyses (Claros et al., 2012; Kyriakidou et al., 2018; Emelianova et al., 2023). While reference-quality assemblies are now available for most major crops, including polyploid species such as wheat, peanut, cotton and mustard, reliance on a single linear reference genome remains a fundamental bottleneck (Thompson et al., 2026). A linear reference genome is typically derived from a single genotype and therefore fails to capture population-level allelic variation, heterozygosity and structural variation (SV). SVs are typically defined as genomic alterations larger than 50 base pairs (bp). Polyploid genomes harbor exceptionally high levels of SV, with variant counts ranging from a few thousand to several million, reflecting their intrinsic genomic complexity and evolutionary dynamism (Zhang et al., 2024; Zhao et al., 2025).

Advances in sequencing technologies now enable the construction of allele-aware and haplotype-resolved genomes, which are particularly important in polyploid organisms (Chen et al., 2020; Zhao and Shi, 2025). Allele-aware references explicitly represent different alleles at a given locus, facilitating the precise identification of individual variants (Chen et al., 2020). Haplotype-resolved assemblies from a single genotype capture the full allelic diversity of heterozygous and polyploid genomes, revealing complex structural features and allele-specific variants (Ryan et al., 2025; Zhao and Shi, 2025; Lei et al., 2026). However, despite these advantages, relying on a single reference genome remains limiting—particularly in polyploid crops, where genome duplication and subgenome divergence contribute to extensive genetic diversity (Hu et al., 2024). A single reference collapses population-level variation and misses non-reference insertions, complex rearrangements, and subgenome-specific haplotypes—key sources of adaptation and agronomic traits (Thompson et al., 2026).

A pan-genome comprises a collection of genome assemblies and their annotated features across multiple individuals. It captures both core and variable genomic components across accessions, enabling the systematic discovery of SVs, presence/absence variations (PAV) and regulatory variants that are often missed by linear reference genomes (Shan et al., 2025; Thompson et al., 2026). Recent large-scale pan-genome efforts underscore the magnitude of this limitation and the necessity of alternative frameworks. Chromosome-resolved wheat pan-genomes have revealed extensive SV landscapes that connect breeding history and habitat adaptation (Jiao et al., 2024), while graph-based pan-genome resources have demonstrated improved trait discovery across diverse environments by reducing reference bias (He et al., 2023). Similarly, pan-genome analyses in potato have shown how transposable elements and ploidy level jointly shape gene content evolution and structural diversity (Bozan et al., 2023). In particular, graph-based pan-genomes provide a powerful means to represent alternative allelic paths and complex rearrangements, improving read mapping in variable regions and enabling more accurate genotyping of structurally diverse loci (Ebler et al., 2022). Rapid advances in graph construction, alignment and visualization tools are also lowering technical barriers to adoption in plant systems (Hickey et al., 2024; Garrison et al., 2024).

Despite these advances, polyploid pan-genomics introduces distinct technical and translational challenges. In allopolyploids, accurate subgenome assignment and haplotype phasing are critical, whereas in autopolyploids, haplotype phasing is particularly complex due to frequent multivalent formation and polysomic inheritance. Additional challenges common to both types of polyploidies include consistent assembly and annotation across accessions, benchmarking of graph-based methods and computational scalability (Fukasawa, 2026). Equally important is the downstream challenge of converting large catalogs of SV into reproducible, interpretable and breeder-ready markers and decision-support tools. Without robust prioritization and validation frameworks, many SVs remain difficult to deploy across breeding programs or to integrate into genomic prediction pipelines (Ko et al., 2022; Hu et al., 2024; Jayakodi et al., 2025; Raza et al., 2026). This review synthesizes recent advances in polyploid pan-genome construction and SVs discovery, critically evaluates linear versus graph-based pan-genome strategies, and highlights the requirements for routine deployment in crop improvement programs. Actionable priorities for the next phase of the field are emphasized, including: (i) the development of standardized, breeder-ready pan-genome platforms; (ii) functional and statistical prioritization of SVs, incorporating machine-learning and AI-assisted ranking approaches; and (iii) integration of pan-transcriptomic and pan-epigenomic layers to interpret dosage effects, regulatory variation, and gene-by-environment interactions. Standardized breeder-ready pan-genome platforms refer to practical analysis systems that allow breeders to visualize genomic variation, identify candidate genes or SVs, and extract markers for routine breeding applications. Such platforms include graph-based visualization tools, standardized pipelines for SV discovery and genotyping, and databases linking SV to phenotypic and environmental information. Importantly, these systems enable the conversion of pan-genome variation into deployable molecular markers and genomic prediction inputs, thereby bridging the gap between pan-genome discovery and routine breeding applications.

**Figure 1:**
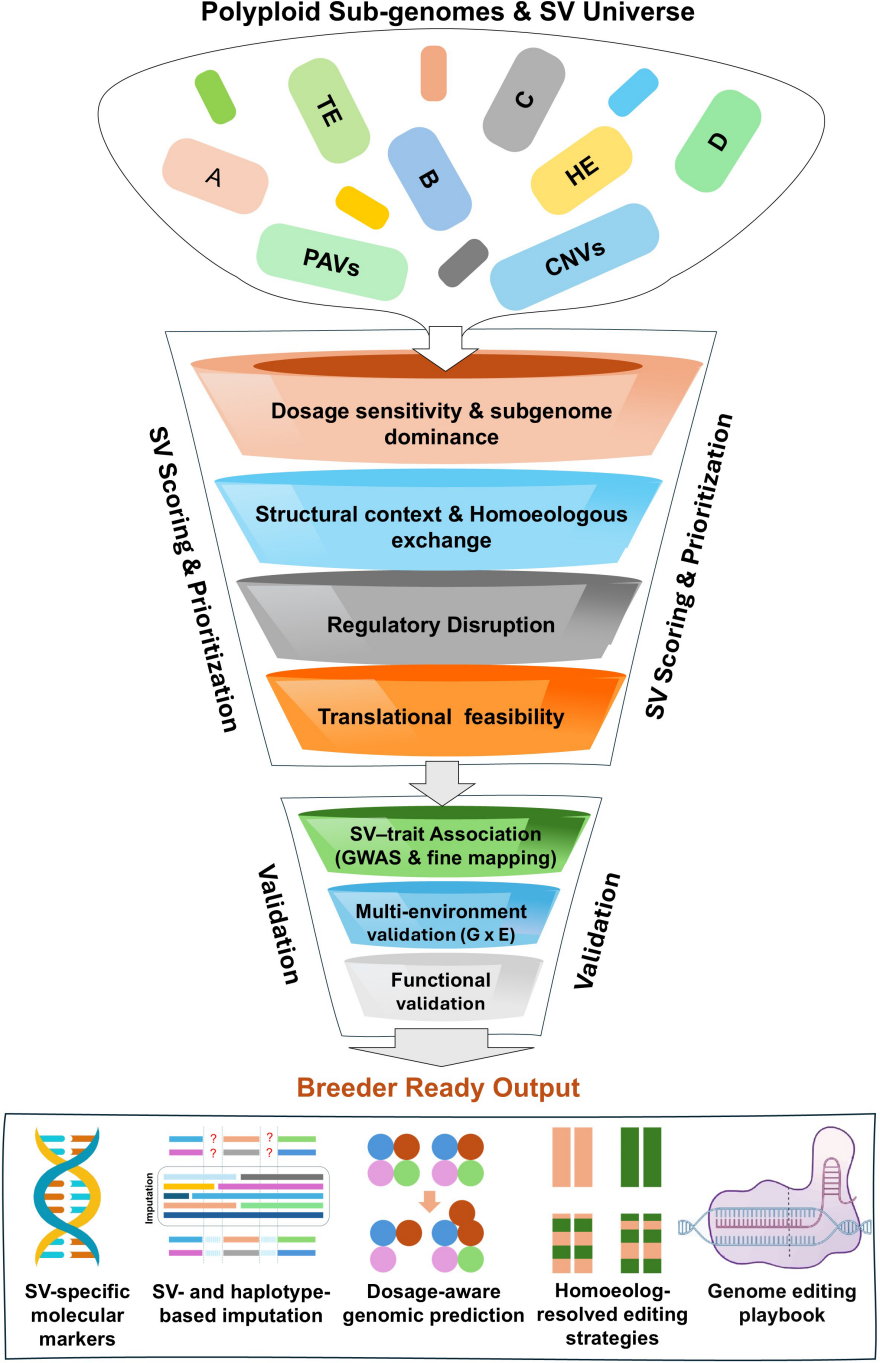
Polyploid structural variation (SV)-to-Breeding Translation Funnel: A polyploid-aware framework for prioritizing SV into actionable breeding outputs. A, B, C, D represent distinct genomes and subgenomes; CNV, copy number variation; G × E, genotype × environment interaction; GWAS, genome-wide association study; HE, homoeologous exchange; PAV, presence–absence variation; TE, transposable element.

Functional and statistical prioritization of SVs is required because pan-genome analyses typically identify very large numbers of SV, only a small fraction of which are biologically relevant. Functional prioritization focuses on variants affecting genes, regulatory elements, gene dosage, or homoeolog balance, or those supported by transcriptomic or epigenomic evidence (**Figure 1**). Statistical prioritization evaluates the strength and stability of SV–trait associations using GWAS, genomic prediction, or multi-environment analyses. Machine-learning approaches such as random forests, gradient boosting, and deep neural networks have been increasingly used to integrate genomic and phenotypic features for variant prioritization. These advances will be necessary to translate polyploid pan-genomics into practical genetic gains in crop improvement. Beyond serving as repositories of genomic diversity, polyploid pan-genomes should be viewed as quantitative genetic infrastructure that fundamentally reshapes how genetic architecture is modeled and exploited in breeding. By explicitly encoding copy-number states, homoeolog dosage and structural haplotypes, pan-genomes enable traits to be decomposed into additive, dosage-dependent and structural components that are invisible in single-nucleotide polymorphism (SNP) centric frameworks. Recent studies in wheat and rice demonstrate that SV and dosage variation explain substantial proportions of phenotypic variance that are otherwise misattributed to residual effects in conventional models, highlighting the need to treat pan-genomes as core analytical substrates rather than auxiliary resources.

## 2 What makes a pan-genome polyploid-aware?

A pan-genome becomes polyploid-aware when it explicitly captures genomic variation across accessions, subgenomes and homoeologs, rather than projecting polyploid diversity onto a single linear coordinate system. In allopolyploid crops, phenotypic variation frequently arises from processes that are either absent or marginal in diploids, including homoeolog dosage imbalance, subgenome dominance and homoeologous exchanges (HE; Bird et al., 2023). These processes reshape genome architecture, alter regulatory landscapes and generate condition-dependent trait variation, yet they are often obscured when pan-genomes are constructed or interpreted using diploid-centric assumptions (Mason and Wendel, 2020; Deb et al., 2023; Jayakodi et al., 2025).

Homoeolog dosage represents a defining feature of allopolyploid genomes and a central requirement for polyploid-aware pan-genomics. Unlike diploids, where gene dosage is largely fixed, polyploids frequently exhibit copy-number variations (CNVs) and PAVs among homoeologous loci, leading to dosage-sensitive shifts in gene expression and phenotype (Lloyd et al., 2018; Schiessl et al., 2019). In hexaploid wheat, genome-wide analyses have demonstrated that variation in homoeolog expression dosage contributes substantially to agronomic trait variation, including yield-related traits, highlighting dosage as a major axis of functional diversity (He et al., 2022). Closely linked to dosage effects is subgenome dominance, whereby one subgenome exhibits preferential gene retention, higher transcriptional activity or greater contribution to adaptive traits. Comparative genomic and epigenomic studies across polyploid plants have shown that dominance patterns are shaped by transposable-element landscapes, chromatin accessibility and cis-regulatory divergence, and can vary across tissues, developmental stages and environments (Burns et al., 2021; Zhang et al., 2024).

Importantly, subgenome dominance in allopolyploids is not merely an evolutionary consequence of polyploidization but has direct implications for breeding, as selection often acts disproportionately on dominant subgenome alleles (Wang et al., 2022). Polyploid-aware pan-genomes should therefore retain explicit subgenome attribution and regulatory context, allowing dominance patterns to be interrogated rather than averaged out. This effect arises when analyses are performed at the whole-genome level without explicitly modeling subgenome identity in allopolyploids, so that expression levels, allele effects, or variant frequencies are summarized across homoeologous loci. When SVs, dosage states, or expression signals are summarized at the gene or locus level without distinguishing between subgenomes, subgenome-specific dominance patterns and regulatory divergence may be underestimated. In allopolyploids, the key biological objective is to understand how ancestral genomes interact within a shared nuclear and regulatory context, and polyploid-aware pan-genomes therefore need to preserve subgenome-resolved information to enable analysis of homoeolog dosage, expression bias and regulatory interactions.

A further requirement is explicit accommodation of HE in allopolyploids. These events transfer chromosomal segments between subgenomes, generating mosaic genomes with altered gene dosage, novel allelic combinations and rewired regulatory contexts. Extensive evidence from wheat, *Brassica* and other allopolyploid crops indicates that HEs are non-random, genotype-dependent and frequently associated with agronomically important traits (Hurgobin et al., 2018; Deb et al., 2023; Coppage et al., 2025). Linear pan-genome representations struggle to consistently model such rearrangements, whereas graph-based pan-genomes can encode alternative subgenome paths and structural configurations, reducing reference bias and improving variant genotyping in regions affected by HE (Eizenga et al., 2020; Hickey et al., 2024).

Finally, a truly polyploid-aware pan-genome must anticipate allele × homoeolog × environment interactions, as polyploid phenotypes often emerge from conditional expression partitioning among homoeologs in response to environmental cues. Recent pan-genome studies integrating environmental and breeding metadata show that SVs and subgenome-specific haplotypes contribute differentially across habitats (Hou et al., 2024), underscoring the need to connect polyploid pan-genomes to multi-environment phenotyping and prediction frameworks (Jayakodi et al., 2025). Together, these considerations define a shift from static, sequence-centric pan-genomes toward dynamic, subgenome-aware genomic infrastructures that are better aligned with the biological reality of polyploid crops and the practical needs of modern breeding.

## 3 Structural variation in polyploid crops

In many allopolyploids, HE arises when chromosomes from different ancestral genomes mispair. These exchanges transfer DNA segments between subgenomes, potentially causing deletions, duplications, or translocations (Mason and Wendel, 2020). Even small, duplicated DNA regions within a genome can occasionally trigger non-homologous chromosome rearrangements. Importantly, HEs represent a major source of SVs in allopolyploid crops, driving their evolution, adaptation and diversification (Hurgobin et al., 2018; Wang et al., 2024). Despite its prevalence, HE is not randomly distributed, it is more frequent in some chromosomes, genotypes and generations than in others (Wang et al., 2024). For example, HE in *Brassica juncea* was found to be asymmetrical, with A-genome segments more frequently replacing C-genome segments (Wang et al., 2024), indicating genomic biases. Although the complete mechanistic underpinning of HE variation is not understood, both global chromosomal processes and local genomic or epigenomic environments are thought to contribute to its occurrence (Zhang et al., 2020). Notably, HE is practically relevant in polyploid crops, especially in allopolyploids such as wheat, canola, cotton, peanut, oat, and banana, where it is becoming linked to important agronomic traits (He et al., 2022; Hao et al., 2025; Avni et al., 2026).

In allopolyploid species, SVs are not only abundant but also intricate due to the presence of duplicated (triplicated or higher-order) genomic regions and homoeologous chromosomes derived from ancestral genomes (Hämälä et al., 2024). Among the most relevant forms of SV in polyploids are genic CNV, where gene segments are present in different copy number across individuals, and PAV, where entire genes or DNA segments may be missing in some individuals but present in others (Yuan et al., 2021; Hämälä et al., 2024). MacNish et al. (2024) developed a multi-species *Brassica* graph pan-genome to explore the effects of polyploidy and SV on genomic diversity. Their study revealed extensive genic PAV, with the polyploid *B. napus* showing the highest number of unique genes and SVs, highlighting the role of polyploidy and SV in genome expansion, functional diversification, and adaptation in *Brassica*.

Recent research indicates that SV contributes more extensively to genomic diversity, often disrupting large regulatory regions (Song et al., 2023; Zhang et al., 2024). Investigations at the gene level have revealed that SV can influence gene expression through both cis- and trans-regulatory mechanisms (Alonge et al., 2020; Zhang et al., 2024). In contrast to SNP, SV often disrupts larger genomic regions, including cis-regulatory elements, making them more likely to cause significant changes in gene expression and phenotypic traits both within and between species (Yin et al., 2020; Pan et al., 2023). For example, a ~25 kb inversion affecting *Aradu.DN3DB/Arahy.5EZV1I* and expression changes in *Aradu.SFU0J/Arahy.VEUG4Z* and *gene.15763* have been shown to influence pod and seed size in peanut (Alyr et al., 2026). Notably, in coding regions, *A. hypogaea* displayed a higher frequency of insertions and deletions in upstream regulatory sequences compared to its wild relative *A. monticola* (Yin et al., 2020). Specific examples include indels (insertions/deletions) ranging from 214 bp to 7.9 kb in peanut, which affect genes involved in flower and fruit development, metabolism, and disease susceptibility, and a 275-bp deletion in *AhARF2-2* that promotes seed expansion (Zhao et al., 2025). In rapeseed, a 1,454 bp insertion in *BnaA03.MAMf* enhances gene expression and increases long-chain glucosinolate accumulation (Zhang et al., 2024). Similarly, in cotton, SVs have influenced speciation, domestication, and crop improvement, including novel variants affecting yield and fiber quality, and a 9 bp indel linked to overcoming reproductive isolation between *G. hirsutum* and *G. barbadense* (Jin et al., 2023). These observations indicate that SV in polyploid crops is not merely more abundant than in diploids, but qualitatively different in origin, scale and functional impact.

## 4 Structural variation detection with pan-genomes

To address the complexity of polyploid crop genomes, researchers have developed several strategies to capture SVs (Bevan et al., 2017). In these approaches, resequencing data from diverse accessions is aligned to a reference assembly and SVs are detected based on deviations from expected read-mapping patterns. Standard analytical techniques include read-depth analysis, paired-end mapping and split-read approaches, which can identify a wide range of SVs (Rausch et al., 2012). Some pipelines combine signals across multiple samples for imputation-based detection (Nguyen et al., 2016). Tools like DELLY integrate short- and long-range reads with split-read data to identify balanced and unbalanced SVs at single-nucleotide resolution (Rausch et al., 2012). While these approaches have been widely used to analyze SV, they have several limitations that should be considered, particularly in the context of complex or polyploid genomes. The most notable is that reference-based approaches are biased against sequences or variations that are not present in the reference genome, which simply cannot be detected, leading to an underestimation of the true diversity (Tao et al., 2019). In allopolyploid crops, such reference-based approaches are further confounded by subgenome asymmetry, where differential gene retention, expression dominance and structural divergence among homoeologous chromosomes can systematically bias SV discovery. For instance, in peanut, the two subgenomes (A and B) show unequal contributions, with B retaining more genes and exhibiting higher expression (Pan et al., 2023). These differences likely reflect post-polyploidization structural and regulatory changes that are poorly captured when SV discovery relies on a single reference genome (Thompson et al., 2026). Additionally, ascertainment bias arises when genetically distant accessions are compared to a single reference, leading to poor read alignment, inaccurate SV calls and systematic underrepresentation of variants in repetitive or structurally complex regions, particularly when short-read sequencing data are used (Mahmoud et al., 2019). By analyzing multiple high-quality genome assemblies from diverse accessions, researchers can identify SV in a more unbiased and comprehensive manner. For example, Guo L. et al. (2025) constructed a haplotype-resolved grapevine (*Vitis vinifera*) pan-genome, revealing genetic diversity, hybridization events, and SVs associated with downy mildew resistance. The pan-genome approach reduces reliance on a single reference, enabling the detection of novel insertions, deletions and other SVs. It is especially beneficial in species with high genetic diversity, complex genomes, or polyploidy. SVs are characterized by comparing entire assemblies or through read alignment strategies that incorporate multiple reference genomes.

## 5 Challenges in assembling polyploid pan-genomes

Generating a genome assembly for polyploid species poses unique and significant challenges due to the complex nature of their genomes (Kyriakidou et al., 2018; Zhao and Shi, 2025). Polyploids possess multiple sets of chromosomes, which increases the difficulty in distinguishing between homologous and homoeologous sequences during genome assembly. This complexity is further amplified in autopolyploids, where the high sequence similarity between duplicated homologous chromosomes often leads to missassembly or collapsed regions (Zhao and Shi, 2025). In contrast, allopolyploids, although generally easier to resolve due to greater divergence between parental genomes, still present challenges in accurately assigning sequences to the correct subgenome (Kyriakidou et al., 2018; Koorevaar et al., 2024). Beyond sequence similarity, a major challenge for polyploid pan-genomes is the lack of standardized assembly and annotation pipelines across accessions, which can introduce systematic differences that are incorrectly interpreted as biological variation. Critically, applying a standardized annotation pipeline across all genomes, even without reassembly provides a practical and effective way to minimize such comparative bias. Additionally, high levels of heterozygosity and the presence of repetitive elements, common in many polyploid plants, hinder accurate contig assembly and scaffolding (Kong et al., 2023). Repetitive sequences, such as nested long terminal repeats (LTRs) spanning 20–200 kb, are common in genomes and often exceed the read length of even long-read sequencing. Similarly, resolving identical interspersed repeats can lead to incorrect joins and the formation of fragmented or chimeric contigs (Kong et al., 2023). These issues are exacerbated when using short-read sequencing technologies, which often fail to span repetitive or structurally complex regions (Wang et al., 2021). Moreover, assembling multiple genomes for pan-genome construction demands consistent and high-depth sequencing coverage across all accessions to ensure reliable detection of SV. The computational requirements also increase substantially with higher ploidy levels, as the number of possible haplotypes grows exponentially (Garg, 2021). Accurate haplotype phasing and subgenome differentiation remain major obstacles in polyploid pan-genomes, as distinguishing true biological variation from assembly artefacts is particularly difficult when collapsed haplotypes, mis-assigned homoeologs and duplicated homologous chromosomes with inconsistent repeat resolution can mimic genuine SVs (Jayakodi et al., 2021). Therefore, assembling a high-quality pan-genome in polyploid species requires integrating long-read sequencing, advanced bioinformatics pipelines and careful experimental consideration to capture the full spectrum of genomic diversity.

## 6 Key considerations in pan-genome construction

### 6.1 Selection of a diverse set of representative genotypes

**Figure 2:**
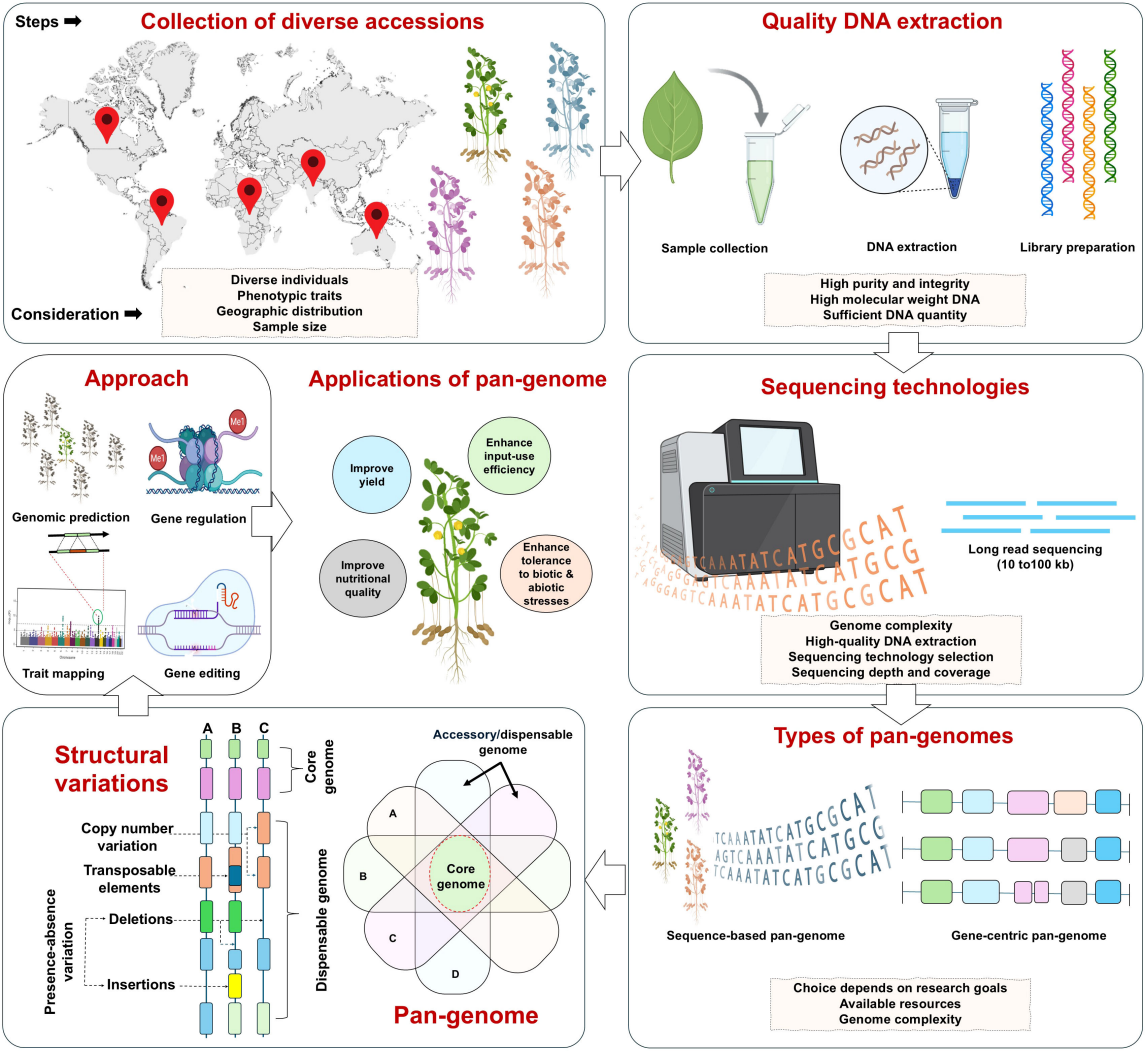
Workflow and key considerations for crop pan-genome construction. The figure illustrates the major steps in constructing a crop pan-genome, from selection of diverse accessions, high-quality DNA extraction, and sequencing, to pan-genome assembly and identification of structural variations (SVs). The pan-genome captures both core and accessory genomes, enabling discovery of SVs that drive phenotypic diversity. These insights can be applied to genomic prediction, trait mapping, gene regulation studies, and precision gene editing to enhance yield, nutrient efficiency, nutritional quality, and stress resilience. Light orange boxes highlight critical considerations at each stage, including sampling diversity, DNA integrity, sequencing strategy, and genome complexity.

The foundational step in developing a robust pan-genome is the careful selection of a genetically diverse set of representative genotypes for whole-genome sequencing and assembly (**Figure 2**). This diversity is essential for capturing the full spectrum of genomic variation across the pan-genome, which can represent variation at the species or even genus level. The objective is to maximize the discovery of novel alleles and variants using a strategically chosen, appropriate number of accessions. The optimal number of genotypes for pan-genome construction largely depends on factors such as genome size and complexity, sequencing cost and the availability of computational resources. For example, constructing an oat pan-genome is far more expensive than a tomato pan-genome due to its larger and more complex genome. Including a larger number of genetically diverse genotypes increases the likelihood of capturing novel SV (Jiang et al., 2024). Inadequate or biased sampling can lead to an underestimation of rare or lineage-specific SV, particularly in allopolyploid crops where subgenome-specific variation may be unevenly distributed across the germplasm. Several strategies are available for selecting core sets in genetic studies (Jayakodi et al., 2021). In many cases, the selection strategy also incorporates trait-specific diversity, aiming to identify SVs associated with key traits. However, trait-driven selection must be balanced with genome-wide diversity, as focusing narrowly on phenotypic extremes can inadvertently exclude structurally divergent but agronomically relevant haplotypes. Genome-wide genotypic data from representative subsets are essential for identifying diverse accessions that capture the full spectrum of germplasm within a species. Using this information (genetic marker data or phenotypic traits data), core sets can be selected through tools like Core Hunter 3 (De Beukelaer et al., 2018), which apply genetic distance-based algorithms to maximize diversity, representativeness and allelic richness. Clustering methods such as principal component analysis and model-based ancestry estimation are widely used in population genomic studies to guide tailored selection strategies (Mascher et al., 2019). These approaches reveal population structure by distinguishing accessions according to domestication history and geographic origin, while also capturing variation in morphological and life-history traits that define breeding gene pools. Much of this diversity is accessed through *ex situ* germplasm collections maintained in genebanks, which serve as primary reservoirs of crop genetic resources (Mascher et al., 2019). The use of such material is increasingly shaped by international frameworks, including the “Nagoya Protocol” which regulates access and benefit-sharing of genetic resources.

Pan-genome panels often include both domesticated and wild relatives. However, genome size varies widely among species (diploids vs allopolyploids), and larger genomes can appear to contain more variants, potentially biasing comparative analyses. In the case of polyploid species, special consideration should be given to include ancestral species or progenitor genomes of polyploid crops. These are key to sussing out the variation inherent from HE and dosage-dependent variation. More distant wild relatives may be added as outgroups to analyze SV and identify novel alleles lost during domestication to improve climate resilience in modern crops (Jayakodi et al., 2021). For example, in peanuts, Zhao et al. (2025) selected eight high-quality genomes (two wild diploids, two wild tetraploids and four cultivated tetraploids) representing variation in seed size and weight to enable the identification of SV linked to these yield-related traits. Similarly, in wheat, 17 cultivars were selected based on their breeding history, grouped into post-2000, 1980s–1990s and 1950s–1960s, to investigate the genomic basis of breeding-driven changes and to identify the key variants that contributed to wheat improvement over time (Jiao et al., 2024). Additionally, in broomcorn/proso millet (*Panicum miliaceum* L.), 32 representative accessions were chosen for *de novo* assembly based on their phylogenetic relationships and geographical distribution (Chen et al., 2023). This extensive sampling strategy demonstrated the power of large-scale genotype inclusion for uncovering valuable allelic diversity and improving functional genomic insights.

### 6.2 Sequencing technologies

Once a diverse set of representative genotypes has been selected, the next critical step is the choice of appropriate sequencing technologies and strategies. High-quality sequencing data is essential to ensure accurate genome assembly, especially for capturing complex genomic regions and SVs. Sequencing technologies, including both short-read and long-read platforms, have transformed genomics by producing large volumes of data in a single run. Pan-genomes can be constructed using both short-read and long-read sequencing data. Short-read assemblies often show reduced completeness and accuracy in complex or repetitive regions due to collapsed sequences, leading to uneven coverage upon read mapping (Wang et al., 2021). In contrast, long-read sequencing offers significant advantages for polyploid crops by enabling the generation of highly accurate, contiguous genome assemblies, essential for resolving complex and repetitive regions common in polyploid genomes (Miller et al., 2017). Long-read sequencing technologies, such as single molecule real-time (SMRT) sequencing, which generates highly accurate high-fidelity (HiFi) reads of ~10 kb or longer, are particularly effective in capturing SVs. This sequencing approach has enabled the development of high-quality pan-genomes in several crops, including rapeseed (Song et al., 2020), soybean (Liu et al., 2020), tomato (Alonge et al., 2020), barley (Jayakodi et al., 2024), rice (Qin et al., 2021) and others. Studies have shown that incorporating ultra-long reads can greatly improve the assembly quality of large and complex polyploid plant genome assemblies (Koo et al., 2023). These reads help recover more repetitive sequences and correct errors effectively. Although earlier long-read platforms exhibited higher per-base error rates, recent SMRT sequencing achieves accuracies exceeding 99.8%, enabling highly reliable assembly and SV detection even in complex polyploid genomes (Wenger et al., 2019).

Combining long- and short-read sequencing data can improve pan-genome completeness and accuracy, particularly when long-read coverage is moderate. Long reads span repetitive and complex regions, enabling accurate SV detection, while short reads can provide additional coverage more cost-effectively, especially for smaller genomes (Miller et al., 2017). Combining these approaches has been successfully applied in several species, including rice (Qin et al., 2021), soybean (Liu et al., 2020), sorghum (Tao et al., 2021) and maize (Hufford et al., 2021), enhancing variant detection and improving genome assembly continuity. By integrating these approaches, researchers can generate more complete genome assemblies and obtain a comprehensive view of genomic variation. In particular, for polyploid genomes, long reads help resolve highly repetitive regions that may collapse in short-read assemblies, preventing underestimation of genic copy number and paralogs. Generally, a depth exceeding 30–50× is sufficient for long-read assemblies to achieve high-quality results, although the required coverage increases with higher levels of heterozygosity, genome repetitiveness, or polyploidy. For instance, in a potato pan-genome study, ten tetraploid genomes were sequenced using 86.3–112.9 Gb of HiFi reads (26–36× coverage per haplotype), resulting in contig assemblies of 2.3–2.7 Gb with contig N50 values ranging from 2.3 to 3.6 Mb (Sun et al., 2025). To improve haplotype phasing accuracy, total depth must be higher to ensure all chromosome copies are adequately covered, because reads are randomly distributed (Jighly, 2021). For example, coverage of 69× for tetraploids, 110× for hexaploids, and 152× for octoploids has been reported to achieve accurate phasing (Jighly, 2021). Nonetheless, the overall cost of sequencing is largely determined by sequencing depth, genome coverage, and the size of the genome. Higher sequencing depth improves data quality and confidence in identifying true genomic variations, making the results more robust and reproducible (Jiang et al., 2024).

## 7 Pan-genomes in polyploid crops

Building upon these foundational efforts, recent advances in pan-genome construction have provided critical insights into the extent of genome diversity in polyploid crops (**Table 1**). The first plant pan-genome was developed in soybean by assembling genomes from seven wild *Glycine soja* accessions, revealing numerous genes absent in the cultivated *Glycine max* genome (Li et al., 2014). Many of these genes were linked to key traits such as disease resistance, seed composition and flowering time (Li et al., 2014). Since then, pan-genomic studies in other crops have uncovered similar trait-associated variations. In peanut (*A. hypogaea* and related species), a pan-genome assembled from eight accessions using Oxford Nanopore and Illumina technologies identified 1,335 domestication-related SVs and 190 SVs associated with seed size and weight (Zhao et al., 2025). More recently, a population-specific peanut pan-genome was developed to better capture genetic variation and recombination patterns that are poorly represented in a single-reference framework, leading to the identification of a unique *AhFAD2C* gene (Thompson et al., 2026). In hexaploid wheat, a pan-genome of 21 accessions revealed genomic changes linked to growth habit via *VRN-A1* (Jiao et al., 2024). In cotton, analysis of 1,807 accessions identified 124 PAVs related to fiber quality and yield (Li et al., 2021). In *Brassica napus*, SVs affecting silique length, seed weight and flowering time were uncovered (Song et al., 2020). These studies highlight the revolutionary potential of pan-genomic approaches in identifying functionally significant SVs, capturing core and accessory gene content, and speeding up trait-based improvement in complex polyploid genomes. Despite these advances, most existing polyploid pan-genomes remain descriptive resources, with limited integration into breeding pipelines, predictive models or decision-support systems. This gap underscores the need to move from cataloguing variation toward operationalizing pan-genomic information for selection and deployment.

**Table 1:** Summary of Pan-genome Construction in Polyploid Crops.

Crop	Species	Accessions	Gene family	Core genome	Accessory genome	Sequencing technology	Key finding	Reference
Wheat	*T. aestivum*	22	1,40,261	–	23.2%	–	Identified key adaptive structural variations	Cheng et al. (2025)
	*T. aestivum*	21	1,70,517	65.6%	27.0%	SMRT and Hi-C	Reported that spring-to-winter shift via *VRN-A1* changes	Jiao et al. (2024)
	*T. aestivum*	16	–	65%	–	Publicly available assemblies	Provides an online visualization tool for the wheat pan-genome	Bayer et al. (2022)
	*T. aestivum*	18	1,40,500	64.3%	35.5%	–	Reported insight into genome diversity in elite wheat	Montenegro et al. (2017)
Cotton	*G. hirsutum*	107	1,08,830	37,728	55,799	SMRT	Novel loci for disease resistance and fiber traits	Yang et al. (2026)
	*G. hirsutum* *G. barbadense*	1,581 and 226	1,02,816 and 79,993	61.8% and 85.8%	38.2% and 14.2%	SMRT	Identified 124 PAVs linked to favorable fiber quality and yield	Li et al. (2021)
	*G. barbadense*	17	54,321	27,337 (50.3%)	19,260 (35.4%)	–	Identified SVs for fiber length, fiber strength and lint percentage	Meng et al. (2025)
Peanut	*A. duranensis, A. ipaensis*, *A. monticola* *A. hypogaea*	8	50,097	34.2%	11.3%	ONT and Illumina	Identified 1,335 domestication-related SVs and 190 SVs associated with associated with seed size and weight	Zhao et al. (2025)
	*A. hypogaea*	8	–	–	–	SMRT	Identified *AhFAD2C* gene encoding fatty-acid desaturase 2	Thompson et al. (2026)
*Brassica*	*B. napus*	8	1,05,672	58,714 (56%)	44,035 (42%)	Illumina, Hi-C and SMRT	Identified causal SVs for silique length, seed weight and flowering time	Song et al. (2020)
	*B. rapa, B. oleracea, and B. napus*	15, 5 and 21	14,43,428	–	–	Publicly available	First multi-species graph pan-genome for *Brassica*	MacNish et al. (2024)
Alfalfa	*M. sativa*	24	54,002	35%	65%	SMRT	Overexpression of *MsGA3ox1* led to a reduced stem–leaf ratio and enhanced forage quality	He et al. (2025)
Cassava	*M. esculenta* and *M. esculenta ssp.* *flabellifolia*	30	33,470	30%	67%	ONT, Hi-C and Illumina	Selective sweeps in genomic regions have domesticated genes	Xia et al. (2025)
Potato	*S. tuberosum*	19	–	40%	2-36%	SMRT and Omni-C	Present a nearly complete pan-genome of autotetraploid European potato	Sun et al. (2025)
	*Solanum* spp	296	1,32,355	17%	82%	SMRT and Illumina	Clade-specific core genes linked to stress response and tuberization	Bozan et al. (2023)
	*S. tuberosum*	44	–	55.4%	7.9%	SMRT and Hi-C	Identified *Soltu.DM.06G025210* gene is key to the initiation of potato tubers	Tang et al. (2022)
Banana	*M. acuminata, M. schizocarpa, M. balbisiana*	15	–	29,331 (61%)	18,359 (38%)	Illumina	Reveals clear genetic differentiation between *Musa* and *Ensete* based on gene PAV	Rijzaani et al. (2021)
Oat	*A. insularis*, *A. longiglumis, A atlantica* and *A. eriantha*	33	–	32%	66%	SMRT and Hi-C	Subgenome-specific expression compensation, structural rearrangements affecting breeding traits	Avni et al. (2026)
Apple	*Malus* Mill	30	4,68,006	–	34,930	SMRT	Revealed major SV and identified a marker for apple scab resistance	Li et al. (2025)
Moso bamboo	*P. edulis*	16	17,38,962	40,302 (53%)	20,963 (28%)	SMRT	Uncovering extensive haplotype-level variation linked to climate adaptation	Hou et al. (2024)
Broomcorn millet	*P. miliaceum*	32	60,015	27,727	24,494	SMRT	Identified 139 loci associated with 31 key domestication and agronomic traits	Chen et al. (2023)

Hi-C, High-throughput Chromosome Conformation Capture; PAV, Presence/Absence Variations; SMRT, Single-Molecule Real-Time; SV, Structural Variation; ONT, Oxford Nanopore Technologies.

## 8 Role of pan-genomics in transcriptomics and epigenomics

Pan-genomes substantially strengthen transcriptomic and epigenomic studies by enabling functional comparisons of expression and chromatin-state variation across diverse accessions, revealing allele- and haplotype-dependent regulation and linking regulatory differences to phenotypic diversity (Raza et al., 2026). In transcriptomic analyses, pan-genomes enable the accurate detection of novel transcripts, alternative isoforms, and expression PAV that arise from gene content diversity and expression variation (Kanwal et al., 2025). Integrating pan-genomic and transcriptomic data further reveals how SV, such as variation in promoter regions, enhancer elements, and sequence variation within gene bodies influences gene expression (Yin et al., 2020; Jin et al., 2023). These SV-induced changes drive genotype-specific expression patterns, contributing to phenotypic diversity in traits associated with adaptation, stress tolerance and development (Zhang et al., 2024). Similarly, pan-genomics advances epigenetic research by enabling the integration of DNA methylation, histone modification, and chromatin accessibility data within a population-wide genomic context. Such integration allows the identification of how SV reshape regulatory landscapes by altering chromatin states and transcriptional activity (Kanwal et al., 2025). Fernandes et al. (2024) mapped crossovers in fully assembled *Arabidopsis thaliana* genomes and found that centromeric regions, though structurally variable, are consistently suppressed for meiotic crossovers. Surrounding low-recombination zones contain retrotransposons and expressed genes, and DNA methylation influences crossover frequency, showing how SV and epigenetic regulation control recombination (Fernandes et al., 2024). Despite these advances, the application of pan-genomes in transcriptomic and epigenetic studies in plants is still at an early stage, highlighting opportunities for further research. One key challenge is that comparing gene expression across diverse lines becomes non-trivial in a pan-genomic context because CNV and PAV change the effective “gene set” being measured; as a result, classic single-gene differential expression frameworks that assume a shared, one-to-one feature space across all samples are not directly applicable. Plant genomics still lacks widely adopted, robust methods for performing expression comparisons at scale under this kind of variable gene content. First headway has been made in barley through pan-transcriptome approaches that explicitly integrate genotype-dependent transcript diversity (Guo W. et al., 2025), and in wheat via *de novo*, cultivar-resolved annotation and pan-transcriptome resources designed to assess gene content, CNV/PAV, and expression differences across cultivars (White et al., 2025).

## 9 Role of pan-genomics in accelerating crop breeding

Modern crop breeding pipelines remain overwhelmingly SNP-centric, an assumption inherited from diploid genetics and reinforced by linear reference genomes. While this framework has delivered incremental gains, it is increasingly misaligned with the genetic architecture of polyploid crops. A central insight emerging from recent pan-genome studies is that SV, homoeolog dosage imbalance and subgenome-specific regulation account for a substantial fraction of adaptive and agronomic trait variation in allopolyploids, particularly for yield, stress tolerance and phenological plasticity (He et al., 2022; Deb et al., 2023). Consequently, SNP-based models systematically underestimate genetic variance, misattribute dosage-driven effects to residual noise and fail to capture the conditional nature of trait expression across environments (He et al., 2022; Jayakodi et al., 2025).

Pan-genomes fundamentally alter this landscape by redefining what constitutes a “marker” in breeding. Instead of treating genomes as collections of independent SNPs, pan-genome frameworks encode PAV, CNV, structural haplotypes and homoeolog-specific alleles as first-class genetic features (Ebler et al., 2022; Jayakodi et al., 2025). This shift exposes a critical limitation of current breeding paradigms: selection models optimized for SNPs are poorly equipped to handle dosage-sensitive, non-additive and structurally complex variation, which dominates polyploid genomes (Deb et al., 2023). As a result, breeding decisions based solely on SNP-derived genomic estimated breeding values risk overlooking high-impact variants that lie outside linear reference coordinates (Ebler et al., 2022).

The implications extend beyond association mapping to genomic prediction. In polyploid crops, yield stability, abiotic stress tolerance and developmental robustness frequently arise from dosage-dependent buffering, subgenome dominance and environment-responsive SVs, rather than from additive SNP effects alone (He et al., 2022; Deb et al., 2023). Pan-genome–enabled prediction models that integrate SVs and CNVs therefore capture genotype × environment interactions more effectively than SNP-only models, particularly under heterogeneous or climate-variable conditions (Bradbury et al., 2022; Vourlaki et al., 2024). For example, He et al. (2023) constructed a graph-based *Setaria* pan-genome and performed large-scale genetic studies across 68 traits in 13 environments, demonstrating that models integrating SVs with SNPs improved prediction accuracy for 73.9% of traits, with some traits exceeding 0.95 accuracy. Similarly, another study demonstrated that combining SNPs and SVs enhanced genomic prediction of phenotypes related to salt stress tolerance and alfalfa quality, with SV-based predictions outperforming SNP-only models in most cases (He et al., 2025). As climate volatility intensifies, the inability of SNP-centric models to represent conditional and structural genetic effects will increasingly constrain predictive accuracy and long-term breeding gains.

Beyond selection, pan-genomes are redefining how genome editing is conceptualized in breeding. Editing strategies designed against a single reference genome implicitly assume sequence uniformity and fixed gene copy number. Pan-genome analyses reveal this assumption to be untenable: effective editing in polyploids requires allele-, copy- and subgenome-specific targeting to avoid unpredictable dosage effects and off-target activity (Tay Fernandez et al., 2022; Jayakodi et al., 2025). For example, pan-genomes allow the identification of core, soft-core, dispensable and private genes across multiple accessions. This information enables the design of subgenome- or homolog-specific guide-RNAs for genome editing by targeting conserved sequences in the intended gene copy while avoiding off-target effects in other homologs or dispensable regions (Tay Fernandez et al., 2022). These findings suggest that polyploid pan-genomes need to be more directly incorporated into breeding workflows. Instead of functioning solely as variant discovery resources, pan-genomes can serve as practical frameworks for detecting structural and dosage variation that can be integrated into selection strategies and genomic prediction models (Jayakodi et al., 2025). In polyploid crops, where structural and dosage variation dominate functional diversity, breeding programs anchored to linear references and SNP-only models will face diminishing returns. By contrast, pan-genome–informed breeding pipelines offer a pathway toward capturing hidden genetic variance, improving prediction under environmental uncertainty and enabling more precise manipulation of complex traits essential for climate-resilient agriculture.

## 10 Challenges and future perspectives

Autopolyploid crops often have highly similar duplicated homologous chromosomes, making it difficult to accurately assemble and distinguish homologous sequences, especially given the often large and repetitive nature of these genomes. Additionally, haplotype resolution in polyploids remains technically challenging, but it is often essential for assigning linked variants to phenotypes (e.g., cis/trans configuration, allele dosage, and haplotype-based association mapping). Genome phasing, a relatively recent approach, allows for the resolution of heterozygous regions and the construction of haplotype-specific assemblies, providing a more accurate representation of complex genomes (Guk et al., 2022). The limited availability of high-quality reference genomes further restricts pan-genome construction and comparative analyses. However, building and analyzing these complex genomes particularly in polyploid plant species requires the development of new algorithms, standard data formats and robust benchmarking tools (Jayakodi et al., 2025).

Over the course of plant evolution, many genes have acquired duplicated copies, either in tandem clusters or dispersed throughout the genome. These duplications often complicate the identification of true orthologs, making gene annotation a challenging task during pan-genome analysis. Errors such as incomplete, missing, or chimeric genes are common, and can lead to misleading conclusions (Jayakodi et al., 2025). As research expands toward broader scales, including genus-level pan-genome analyses, consistency in genome assembly and annotation pipelines becomes essential for ensuring meaningful comparisons. Under such standardized frameworks, cross-species pan-genomic analyses are central to advancing both fundamental plant biology and applied crop improvement.

Crop pan-genomics, especially in polyploid species, remains in its early stages, with significant potential to uncover genome diversity and its functional relevance. By capturing variation across both cultivated and wild accessions, pan-genomes provide valuable insights into the genetic basis of plant evolution, domestication and gene flow. Moving forward, pan-genome research will increasingly integrate transcriptomic and epigenomic layers to better understand how SV, PAV, and regulatory elements influence gene expression and trait development. An important future direction in polyploid pan-genomics is the integration of SV with genotype × environment analyses. Large-scale pan-genome analyses in polyploid crops have demonstrated that the frequency, distribution and functional effects of SV and subgenome-specific haplotypes are strongly structured by environmental gradients rather than being uniformly distributed across populations. In wheat, climate-associated SVs affecting flowering time, stress signaling and developmental plasticity were shown to underpin regional adaptation and breeding-driven selection, highlighting the role of pan-genomes in resolving environment-responsive genetic architecture. More broadly, comparative analyses across plant systems indicate that polyploid genomes exploit dosage variation, homoeolog expression partitioning and regulatory SVs to buffer environmental stress and enhance phenotypic plasticity under fluctuating climates (He et al., 2022; Jayakodi et al., 2025). Integrating pan-genome variation with multi-environment phenotyping and environmental metadata therefore represents a critical step toward predictive breeding frameworks capable of identifying climate-resilient haplotypes and SVs for future agroecosystems.

Pan-genome analyses have increasingly highlighted that genomic variation alone is insufficient to explain phenotypic diversity in polyploid crops, necessitating the development of pan-epigenomes that capture heritable variation in DNA methylation, chromatin accessibility and histone modifications across accessions (Jayakodi et al., 2025). In polyploids, copy-number variation, PAV and HE frequently alter gene dosage; however, their phenotypic consequences are often buffered through epigenetic mechanisms that modulate transcriptional output, a process broadly referred to as dosage compensation (He et al., 2022; Deb et al., 2023). Empirical evidence from allopolyploid systems demonstrates that subgenome-specific DNA methylation and chromatin state asymmetry contribute to expression dominance, selective gene silencing and conditional activation of homoeologs under stress or developmental cues (Burns et al., 2021). Integrating epigenomic layers into pan-genome frameworks, therefore, enables the distinction between structurally equivalent but epigenetically divergent alleles, revealing why identical SV or CNV can exhibit contrasting functional effects across genotypes and environments. As emphasized in recent syntheses, pan-epigenomes represent a critical next step for translating polyploid genomic diversity into predictive models of gene regulation, phenotypic plasticity and climate resilience (Jayakodi et al., 2025).

Developing pan-transcriptome and pan-epigenome atlases across diverse accessions will enable more precise identification of causal variants through integrative analyses. This knowledge can help reveal the mechanisms behind cross-incompatibility within gene pools, one of the major barriers to utilizing wild relatives in breeding programs. Coupling these discoveries with precision tools such as CRISPR/Cas gene editing enables direct modification of key genes or regulatory regions, while genomics-assisted breeding can harness pan-genome-derived variants to accelerate the selection of desirable traits. Together, these multi-omic and breeding innovations hold great promise for developing high-yielding, stress-resilient polyploid crops.

## 11 Conclusion

Polyploid pan-genomics marks a decisive shift from static, linear references toward dynamic, subgenome-aware representations of crop diversity. In complex polyploid crops, linear genomes are no longer sufficient to capture the structural, regulatory and dosage-driven variation that underpins adaptation and yield stability. Pan-genomes—particularly graph-based and multi-omic extensions—are rapidly becoming essential infrastructure for breeding, enabling the systematic discovery, prioritization and deployment of SVs under diverse environments. As climate volatility intensifies, translating polyploid pan-genomic diversity into predictive and breeder-ready platforms will be critical for sustaining global food security. In polyploid crops, where structural and dosage variation drive adaptive potential, pan-genomes are foundational infrastructure for breeding systems capable of operating under climate uncertainty.
